# Mount Vernon Hospital for Consumption

**Published:** 1905-06-17

**Authors:** 


### MOUNT VERNON HOSPITAL FOR CONSUMPTION.

### A RECORD COLLECTION.

The Mount Vernon Hospital for Consumption as the result of its festival dinner, held at the Hôtel Cecil, on the 8th inst., had a record collection—£12,721 was the total received through the dinner committee's efforts, or over £1,500 more than the previous record of the hospital, viz. £11,000. The President's list alone totalled £5,500.

The Marquis of Zetland, the president of the hospital, who occupied the chair, in proposing "The Future Prosperity of the Mount Vernon Hospital for Consumption," said that whilst he did not pose as an expert in the diseases which attacked the human frame yet he did know something of the work done by that hospital. As President he had visited it on many occasions, and as a subscriber to its fund he had for some years now enjoyed the privilege of sending patients to its wards from Yorkshire and from his own estates in that county. The story those patients told him on their return was always to the same purpose. They were one and all appreciative of the great kindness they had met with at the hospital, appreciative of the comforts they had enjoyed there, and thankful for the benefits they had derived from the treatment extended to them. (Applause.) Yorkshire, however, was not the only county which had felt the benefits of that institution—there were few parts of the United Kingdom which had not been helped by it. (Applause.) Starting as a small local institution for the northern part of London, it now consists of two hospitals — one at Mount Vernon with 145 beds, and the other (opened in 1904) at Northwood with 110 beds—and an out-patients' department in Fitzroy Square. Unfortunately, the committee were unable to open more than 140 out of the 250 beds, leaving 110 unutilised for want of funds, although the hospital had 360 selected cases on its books waiting for admission. The committee, therefore, were appealing for £100,000 to clear off the debt on the hospital, and to place its finances on a sound basis.

Mr. Henry Stedall (chairman of the Committee of Management), in replying to the toast, deeply regretted the loss the hospital had sustained in the death of Mr. Benjamin A. Lyon, who, for 20 long years, amidst the stress and strain of difficulties, had fought the battle of that hospital. (Hear, hear.)

